# Path Loss Measurements and Model Analysis in an Indoor Corridor Environment at 28 GHz and 38 GHz

**DOI:** 10.3390/s22197642

**Published:** 2022-10-09

**Authors:** Tolulope T. Oladimeji, Pradeep Kumar, Mohamed K. Elmezughi

**Affiliations:** Discipline of Electrical, Electronic and Computer Engineering, University of KwaZulu-Natal, Durban 4041, South Africa

**Keywords:** 28 GHz, 38 GHz, indoor environment, line of sight, millimeter wave, non-line of sight, path loss, path loss models, wave propagation

## Abstract

This paper examines the large-scale path loss models for an indoor corridor environment at frequencies of 28 and 38 GHz. The measurement environment consists of an indoor corridor with both line-of-sight (LOS) and non-line of sight (NLOS) scenarios using vertical–vertical (V–V) and vertical–horizontal (V–H) antenna polarizations. The single-frequency close-in (CI), floating intercept (FI), free space large-scale path loss models and measured data from the measurement campaign were used to evaluate the performance analysis. The paper also focuses on various parameters, such as standard deviation, path loss exponent (PLE), accuracy, simplicity, and stability of the models. The analysis focuses on the peculiarity of the effect of the wall proximity on the path loss parameters as well as comparisons with the parameters in some of the reviewed literature studies. The FI and CI models produce comparable results for both antenna polarizations and clearly fit with the measured path loss. The PLE, with the highest value of 3.33 at 38 GHz (V–H), is much higher in the NLOS scenario with V–H polarization due to the signal degradation along the path from the transmitter (Tx) to the receiver (Rx). This is because there is no direct LOS between the Tx and Rx antennas. The Rx only relies on signal diffractions and reflections from obstacles as it transmits through the path from the Tx antenna. The path loss measurements and model analysis presented here are useful in designing 5G wireless communication systems for indoor environments, particularly for power budget calculations.

## 1. Introduction

High data rate wireless communications are essential, and a number of solutions have been presented to address this issue. The requirements for greater capacity, high dependability, data rates, and quality for a number of applications have been met by a variety of current wireless communication systems, notwithstanding this. The most solid answer to this issue is millimeter wave propagation. It is referred to as centimeter wave (cm-wave) or millimeter wave (mmWave) spectra. Fifth-generation (5G) technology has drawn interest since it can provide data rates of many gigabits per second (Gbps) [1,2]. The lower band has been used in many wireless systems, whilst the majority of the upper portion of the spectrum is not used (but may be used) for 5G technology. Potential commercial uses, such as automobile radars and high-data-rate systems, have been made possible due to the rising demand for inexpensive circuitry and better data transfer rates in these bands [3,4,5]. Achieving complete transparency and rate convergence between wireless and wired links, millimeter wave (mmWave), and terahertz (THz) bands, which are the new wireless communication technology frontiers, could lead to seamless interconnections between ultra-high speed wired networks (fiber optic links) and personal wireless devices (laptops). 

**Figure 1:**
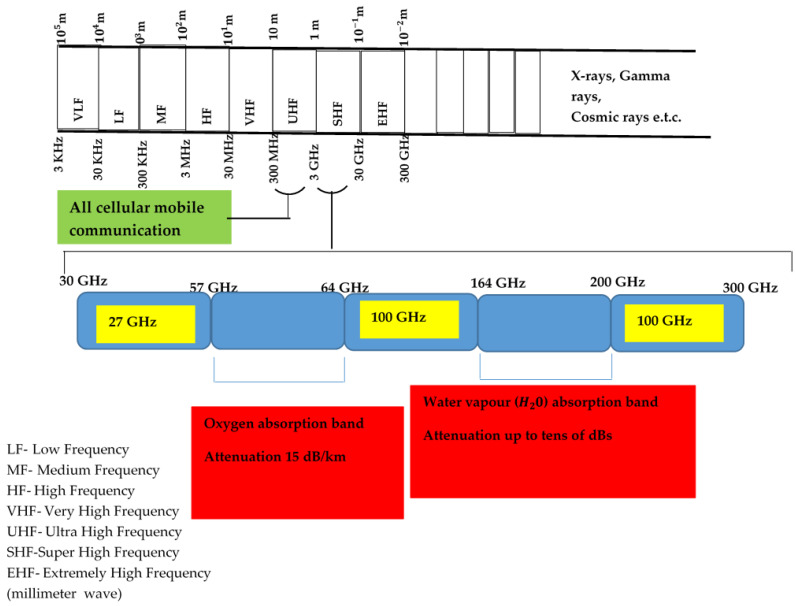
Millimeter wave spectrum [12].

Millimeter wave technology operates in the electromagnetic range between 30 and 300 GHz, with wavelengths ranging from 10 to 1 mm [6,7,8,9,10,11]. A detailed diagram of the millimeter wave spectrum is shown in Figure 1. The available spectrum at the presented frequencies is more than 200 times larger than all of the present cellular network allocations [7]. This technology is characterized by a large quantity of idle bandwidth that can improve the data rate available to end users, allowing it to meet the two primary requirements for fifth-generation (5G) networks: ultra-high peak throughput (20 Gbps) and the average user experience rate (50–100 Mbps) [9]. Since gigabit Ethernet and desktop connections have become inexpensive for server connections, in 2007, the communication sector disclosed the unoccupied 10 Gb for usage [9,10]. In addition, gigabit Ethernet became the standard for servers, requiring systems to be ordered with gigabit network interface cards on a regular basis [10]. Over time, the cost of wireless gigabit lines has equaled the cost of wireless. This provides superior output in older wireless applications as well as other feasible uses at gigabit speeds. Wireless communications have become increasingly relevant in the business world, particularly in USB (Universal Serial Bus) 2.0, gigabit speeds, and extended range connectivity, with significant applications in high-quality multi-media, phones, and data services [10].

Previous wireless local area network (WLAN) speeds were just 54 Mb/s, with IEEE 802.11n applications reaching 150–300 Mb/s. However, when it comes to accessing rich media information, even 500 Mb/s is insufficient. In the near future, home A/V (audio/video) networks will need Gb/s data rates to deliver uncompressed high-definition video at resolutions of up to 1920–1080 progressive scans, with latencies ranging from 5 to 15 ms [10,11]. In addition, the technical requirements for high-speed wireless systems must take into account the following factors:

1. The requirement for higher data rates will continue to grow as the demand for multimedia networks grows.
2. The demand for shared resources has increased as a result of data streaming for both personal and mobile devices.

Although numerous technologies have been used, such as IEEE 802.11n, IEEE 802.16 (WiMax), and ultra-wideband (UWB), their efficiencies have not been sufficient to meet the demands placed on wireless communications, particularly in fifth-generation (5G) networks [11]. A better way to tackle these challenges is to make proper use of frequencies that are not employed in millimeter waves but have application potential. These frequencies are shown in Figure 1 [12]. Despite the fact that millimeter wave technology has been in use for some time, with the advent of process technology, this technique has begun to receive widespread acceptance among academics and industries.

After building a communication channel that spans the capacity and dependability of data rates, the path loss exponent is one major metric that a good communication channel depends on [1,4,13,14]. Path loss is a measure of the degeneration of the propagated signals over a distance range in both line-of-sight (LOS) and non-line of sight (NLOS) scenarios. The path loss exponent is used to evaluate the performance of a communication system in wireless channel propagation. It has been found that impediments, including walls, furniture, and people, can cause propagation loss in millimeter wave technology. The development of a good path loss prediction model for these bands still depends on detailed characterization, analysis, and modeling in these frequency bands, despite the fact that research in this field is ongoing. Wireless system design, planning, and simulation were all part of the model application. Different path loss model concepts have been proposed by various academics for examination, particularly in indoor environments [15,16,17,18,19,20,21]. The most important use of path loss models will be in [18], where calculations of power budgets, modulations, forecasts of cellular coverage/interferences, and the design of coding schemes are all highly critical.

This study, which offers LOS and NLOS measurement data scenarios in an indoor corridor environment, assesses CI and FI path loss prediction models for frequency ranges of 28 and 38 GHz. The measuring campaign took place on the 5th floor of the Discipline of Electrical, Electronic, and Computer Engineering Department building at the University of KwaZulu- Natal’s Howard Campus in Durban, South Africa. In the NLOS scenario, the evaluation included propagation characteristics for both FI and CI free space path loss models. Oyie and Afullo [22] worked on the same channel settings of 14 and 22 GHz using the Rohde & Schwarz SMF 100A for signal generation at the transmitter (Tx), Rohde & Schwarz FSIQ signal analyzer at the receiver (Rx), and two directional pyramidal horn antennas. Previous work of [18] did not consider the behaviors of the model parameters when two antenna polarizations were used but dwelled only on one antenna polarization, which was the V–V antenna polarization in an indoor environment. However, to fill this gap, this paper evaluated the measurement analysis of the existing CI together with the FI path loss models in terms of the antenna polarizations using measured data from the measurement campaign. The measurement campaign was conducted at frequency bands of 28 and 38 GHz using the Rhode and Schwarz SMB 100A radio signal generator to generate continuous wave (CW) signals, which were then delivered across a wireless medium to the Rhode and Schwarz FSIQ 40 signal analyzer of a frequency range of 100 KHz to 40 GHz with the use of broadband horn antennas at both the transmitter and receiver ends. The accuracy, simplicity, and stability of the parameters of the models were evaluated in both LOS and NLOS scenarios.

This work established an appropriate antenna polarization mode with the lowest path loss when transmitting power from the transmitter to the receiver in both LOS and NLOS communication scenarios. It will also be a good indication for developmental initiatives for future wireless communication research on specific building design prediction path loss models for brick and concrete walls (since the indoor corridor is made of dry concrete and bricks). The rest of the paper is organized as follows: Section 2 reviews the literature studies and works on millimeter waves. Details of the environment used for the measurement campaign and various path loss models are discussed in Section 3. The results and the comprehensive discussion of the results are presented in Section 4. The concluding remarks are presented in Section 5.

## 2. Literature Review

We review heavy path loss, wide bandwidth, narrow wavelength, and high penetration that characterize millimeter wave communication. These features are mentioned as follows:

(a) Abundant bandwidth: At the moment, the overall bandwidth available for a mobile network is insufficient to meet the increased data demands of devices (in fact, the available bandwidth is less than 780 MHz for 2G, 3G, and 4G networks). The most significant advantage of millimeter communication over classical communication is the increased bandwidth, which allows the transmission at frequencies exceeding 150 GHz [15].
(b) Short wavelength: Because mmWave signals have wavelengths in the order of millimeters, they must be communicated using MIMO, and they are particularly well suited to packing a large number of half-wavelength-spaced antennas into tiny spaces. The combination of mmWave with massive MIMO can considerably improve wireless access and throughput performance [23].
(c) Propagation loss: Path loss and penetration loss are two different types of propagation losses. Under the assumption of the line-of-sight (LOS), the free space route loss is proportional to the square of the carrier frequency, according to the Friis transmission formula. Because microwave frequencies start at 26.5 GHz, there is more propagation loss than in the microwave band. For example, at 60 GHz, it is 28 dB higher than at 2.4 GHz. This has been a key drawback of mmWave, but with the introduction of device to device (D2D) communications, a high-gain directional antenna may compensate for the loss, improving network capacity and enhancing security against eavesdropping and jamming. The increased penetration loss in NLOS settings makes it difficult for mmWave nodes placed outdoors to reach indoor spaces. Signals/propagation may suffer from large penetration losses in the case of indoor users with outdoor base stations (BSs), lowering data throughput, spectrum efficiency, and energy efficiency. The separation of outside and indoor scenarios is, therefore, unavoidable [24,25].

According to Salous et al. [26], there are unallocated spectra in millimeter wave bands, preventing the full use of massive antenna arrays for high-speed data transfers. Although gigabit data transmissions in these bands necessitate accurate channel modeling, the shadowing effect and the necessity for adaptive beam formations in areas with significant mobility persist. As a result, it was suggested that in addition to end-channel sounders, detailed measurements for full radio characterization should include angular spread and delay time in the evaluation of multipath make-ups. However, in order to provide input to standard organizations, the lack of channel models must be addressed. El Hajj et al. [27] emphasized the relevance of millimeter bands as a proven solution for high data rate transmissions, particularly in indoor environments. However, millimeter wave propagation technology has been reported to suffer from propagation loss of 25 to 30 dB due to impediments, such as walls, furniture, and human blocking. Another study used a frequency domain and a vector network analyzer (VNA) to investigate millimeter wave propagation at 60 GHz in an indoor environment. The results suggest that an access point (AP) can be placed in the center of the network to reduce shadowing caused by human impediments. It has a high frequency that could be solved with a frequency lower than 60 GHz.

As mentioned in [28], millimeter wave measurements were made in China with the goal of determining the influences of atmospheric variables on transmission. There was a difference between the theoretical rain-induced signal attenuation and the practically recorded signal attenuation during the rainfall. It was founded on the idea of rain forecasting and monitoring in real-time. As a result, the dynamic rain-aware link adaptation method was developed to allow the system to fit the modulation and coding scheme to rain intensity levels, the production efficiency of fixed modulation, and coding schemes. The results reveal that the rain-induced signal is unpredictable in both practical and theoretical situations, with attenuation due to variations in weather conditions ranging from 1.5 to 4.5 dB. The drawback was that the different links were not compared. Gade et al. [29] discovered that on-chip wireless links function better than a standard network on a chip (NoC) in additional millimeter wave research. The on-chip wireless channel characteristics were combined with antenna implementation and near-field multipath propagation effects. The near-field/transition region is where the propagation in the on-chip wireless channel takes place, making the channel more difficult. On antennas, it was also discovered that directional kinds are less impacted by channel time dispersion, despite higher losses, and that omnidirectional antennas obtained a reserve. The on-chip wireless channel provides information on the characteristics of wireless communications and aids in the design of circuits for improved performance.

The performances of millimeter waves for indoor communication at different bands between 28 and 73 GHz were carefully examined for LOS and NLOS conditions, taking into account the effects of different buildings and frequency-sensitive materials. The links between separation distances and the received power and delay spread were inversed. As a result of the concerns with the antenna’s directivity, a rise in path loss causes a commensurate increase in separation distance. The system was also able to solve the problem of bandwidth in electronic devices, allowing for the growth of low-cost infrastructure demand for broadband mobile devices. The main disadvantage of this strategy is that it tends to fail as distance and communication capabilities grow [30,31,32]. Chittimoju and Yalavarthi [33] conducted a complete assessment of millimeter wave communications, including some of the benefits and uses. They demonstrated that millimeter waves encourage larger bandwidths while increasing the speed to 10 Gbps. Some of the benefits observed include the utilization of compact components, less interference, and increased security. The range is limited in the line of sight, which was one of the key flaws uncovered. The authors in [34] introduced a novel technique termed the Q learn-based system, which incorporates the edge computing function in adjustable power and angle sub-6 GHz user equipment to tackle capacity and efficiency problems in millimeter wave propagation. The results suggest that the user’s equipment (using this method) was able to achieve great energy efficiency, allowing for a very strong and steady transmission capacity.

Maltsev et al. [35] concentrated on the merits, limitations, and common applications of millimeter wave communications in several bands. Millimeter wave was determined to be critical in the deployment of 5G, and it is believed that significant improvements in radio and networks could be developed to aid in the deployment of 6G. Fuschini et al. [36], using ray tracing (RT) simulations and directed measurements, investigated the narrowband and wideband properties of an in-room 70 GHz wireless channel. Reflection is the most pronounced mode of propagation; however, scattering is still present and appears more than when the frequencies are below 6 GHz, according to the observations. When comparing a more detailed environment to a less detailed environment, if both were exposed to the same error sources, a faster rate of calculation was seen, but this did not translate to greater simulation accuracy.

In [27], the authors reviewed the results of observations taken in two indoor locations at a transmission frequency of 60 GHz. The responses of three distinct types of antennas to power loss factors were verified using a vector network analyzer (VNA). The results revealed that big aperture antennas had greater guided wave effects than those with narrow apertures, resulting in a more accurate path loss model for the latter. The measurements also showed that positioning an omnidirectional antenna in the access point (AP) in the center of the meeting room provided better radiation than placing it in a corner because the shadowing effect produced by human obstruction was reduced. These findings will aid in the deployment of new wireless local and personal area networks. Further research work included compiling a comprehensive overview of 5G network approaches in millimeter wave wireless communication systems, as well as bringing together essential millimeter wave propagation models from the past to the present. It also emphasized the significance of developing diverse models based on ray tracing and measuring procedures for current and future uses in academia and industries. As millimeter wave is still in the research era, especially in the application for 5G propagation [37], the data acquired on shadowing and path loss will aid in the predicted improvement. The major issues with millimeter wave propagation, such as limited beam width, high penetration loss, and strong path loss, were highlighted in [25]. The authors also discussed the differences between analytical modeling and ray tracing approaches for channel modeling. After the measurements, data processing and analysis of the measurement results were given, including channel gain, scatterer identification, RMS delay spread, and average power delay profile (APDP). When taking measurements in varied settings, however, the usage of a MIMO channel with a wide frequency spectrum is essential.

Elmezughi et al. [19] discussed the frequency measurements in 14, 18, and 22 GHz frequency bands in an indoor environment. The authors of [19] also presented two path loss prediction models and communication scenario analyses for both NLOS and LOS. The LOS analysis showed that the CI and FI models functioned nearly identically after execution at all frequencies. With the frequencies increasing along the LOS range, the PLE increased significantly in the CI model. It rose from 1.37 to 1.66 for the 14 and 22 GHz frequency bands, respectively; however, the LOS values did not match those of the FSPLE. Due to environmental considerations, the path loss models had symmetrical features at around 180° AoA. The models, on the other hand, performed better at 30°, 330°, and 180° AoA. The findings also demonstrated that CI and FI models may be employed reliably in both LOS and NLOS corridor scenarios. The main flaw that needs to be addressed is the adoption of a higher gain antenna to decrease additional path losses to the absolute minimum. In addition, as an extension to the above-mentioned conclusion, the effects of transmitting antenna heights on these parameters were explored in [20]. Elmezughi and Afullo [21] recently updated this work, delivering efficient improvements for both the CI and FI path loss models. The major findings showed that, for both LOS and NLOS communication scenarios, the enhanced models beat the standard models. Furthermore, the proposed models have substantially superior stability and sensitivity than standard models, especially in the NLOS condition. By combining these enhanced models with the LOS probability models provided in [24], a generic and accurate model for indoor corridor environments may be obtained. Despite the research on these frequency bands, thorough characterization, analysis, and modeling in these bands remain crucial. This study used the MMSE approach to analyze large-scale path loss models in the 28–38 GHz frequency spectrum in order to reduce the complexities and errors in LOS and NLOS scenarios.

Path loss is a phenomenon that occurs when a transmitter’s signal is attenuated in the communication channel as a function of the distance traveled as well as the propagation channel characteristics. Path loss (or path attenuation) is the decrease in the power density of an electromagnetic wave as it travels through space. Path loss can be caused by a variety of factors, including natural radio wave expansion, diffraction path loss caused by obstruction, and absorption path loss due to the presence of a form of media that is not transparent to electromagnetic waves. It is crucial to remember that even when a path is lost, the transmitted signal may still travel along other paths to its destination; this process is known as multipath. Since these waves or transmitted data travel along different paths, the wave may reconvene at the destination point, resulting in significantly different received signals [38].

It also refers to the loss or attenuation that a propagating electromagnetic signal (or wave) experiences as it travels from the transmitter to the receiver. As a result, the received power is lower than the broadcast power level. Antenna gains, operational frequency, transmission power, and the range of separation between the transmitter and receiver are all elements to consider. The most common way to express path loss is in decibels (dB) [39]. Because the dependency of the distance between the transmitter and the receiver distance on the path loss is no longer linear, the path loss in wireless propagation is mostly a function of a logarithm factor. However, in LOS environments, signal attenuation over distance closely follows Friis’ free space path loss equation, propagating signals attenuated according to the square power law [39,40].

In the last 20 years, there has been substantial research into various propagation channels that could be used for indoor channels. While some have concentrated on both outdoor and indoor office spaces, others have concentrated solely on interior office environments [41,42,43,44,45,46,47]. Wang et al. produced model descriptions with probability distributions; they relied on the parameters in research on an empirical path loss model for wireless channels in indoor short-range office environments. The model depicted appreciable variable values of route losses at different frequencies and produced a simpler model that simplified radio propagation in difficult situations. However, because this study was conducted in an office setting, it was necessary to evaluate this innovative prediction path loss model in a commercial setting with more obstacles [48].

Further research has revealed that most propagation models that work at frequencies less than 6 GHz are inapplicable when considering route loss models for millimeter wave frequency bands (which are generally above 6 GHz). Majed et al. constructed channel models that could operate in indoor circumstances at frequency ranges of 4.5, 28, and 38 GHz in order to find a solution. Both LOS and NLOS measurements were taken in an inside office environment with the transmitting and receiving antennas separated by 23 m. The purpose of the study was to compare the new large-scale generic path loss models to existing path loss models, omnidirectionally and directionally, as well as multi-frequency and single-frequency path loss models. The investigation shows that when modeled with one parameter path loss exponent (PLE) and associated with transmitted power, the large-scale route loss model has a propensity to perform better [48]. Shadowing and attenuation, which were explored in [49], are another set of properties common to indoor environments. Wireless open-access research platform (WARP) equipment was used to model path loss and shadowing. As a result, the propagation path loss value was consistent with measurements in the literature, with an exponent of 4 and a standard variation of 6.4 dB.

In millimeter wave propagation, the direct exchange of information between two near-distance devices in the absence of a base station (known as D2D, or device-to-device communication) has various advantages, including energy efficiency, increased data throughput, and shorter latency [31,50]. The effect of path loss on this D2D communication, however, is unique and unsurpassed. Modeling a method that will result in a significant reduction in attenuation is required. The authors in [51] proposed a strategy that uses mode assignment by reuse as well as cellular mode dedication based on a tradeoff of path loss attenuation and ranges between D2D users. This scheme’s analysis was compared to other existing schemes, such as the alternate offer bargaining game theory algorithm (AOBG) and the heuristic algorithm. The main benefit of the proposed approach is that the D2D user’s SINR threshold is supported to a certain extent. The practicality of this technology is demonstrated by the fact that it is extremely useful in situations where path loss attenuation is a concern in both indoor and outdoor contexts. Another measurement work was conducted in two different locations in the United States of America (USA) by MacCartney. Jr. et al. to check the path loss models for 5G millimeter wave propagation channels in urban microcells using the best of the sliding correlator channel sounder at two frequency bands (28 and 38 GHz). Using directional antennas of varying heights and gains, this experiment investigated multiple microcellular conditions. The linear regression fits were used to create the path loss models. The path loss spanned a distance that depended on the power received, according to the measurements. When compared to existing path loss models, the suggested model performed better in terms of lowering shadow factors by several decibels and providing a better fit to empirical data while permitting only a minor path loss [52].

Naruke et al. proposed an indoor localization method based on the path loss—distance relationship using handset sensor data. This proposed model computed the range between the Bluetooth low energy (BLE) transmitter and the smartphone by first using the distance and path loss relationship and then using (PDR) pedestrian dead-reckoning fixed on the mobile phone’s accelerometer. When compared to previous methods, the results revealed a significant improvement in the distance error [53]. Al-Saman et al. conducted a comprehensive investigation in which the investigated mmWave propagation models as well as measurements in indoor environments. Time dispersion and path loss were identified as the key indoor wireless channels in terms of millimeter wave propagation. Although the path loss coefficient grew as the frequency increased, the exponent was only affected by the structure and kind of environment, not by the frequency. The overall observation is that CI and FI models are the best for both LOS and NLOS channel propagations in millimeter wave bands, especially in indoor environments, based on multiple applications of different research articles regarding frequency ranges of 28 to 100 GHz. This development represents a significant step forward in the deployment of millimeter wave propagation for both 5G and 6G networks with low propagation losses [17]. To the best of our knowledge, there is no other analysis that covers the peculiarity of the effect of the proximity of walls on path loss parameters. To fill this research gap, one of the main goals of this work was to find an acceptable model with the best line of fit and the simplest application for the path loss model estimation in both LOS and NLOS scenarios. The well-known single-frequency CI and FI path loss models for V–V and V–H antenna polarizations were used to examine the channel characterization. The work also shows that the multipath components added up favorably due to wave-guiding and reflections in the inside corridor environments. Another contribution from this investigation is that the proximity of the walls, the materials of the walls, the floor, and other irregular elements, such as wooden doors, concrete, and elevator doors along the corridor, all have effects on radio wave propagation (indoors).

## 3. Path Loss Measurements and Models

This section presents the details of the environment used for the measurement campaign and the propagation models.

### 3.1. Measurement Setup

**Figure 2:**
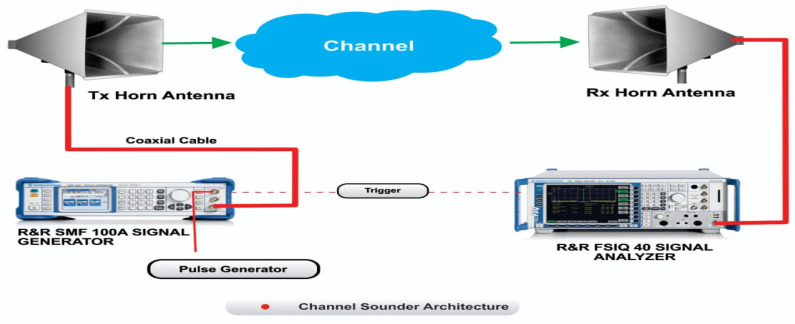
The architecture of the channel sounder.

The setups used for the measurement, the measurement scenario, and the channel sounder are shown in Figure 2. The measurements were carried out at the Howard College Campus of the University of KwaZulu-Natal on the 5th floor of the Department of Electrical, Electronic, and Computer Engineering. The measurements were conducted at frequencies of 28 and 38 GHz with a transmitting antenna height of 1.6 m and receiving antenna height of 2.3 m. There was a total of 13 measured points for the both LOS and NLOS scenarios. Although a reference distance of 1 m was observed, subsequent measurements were done from 2 m points with increments of 2 m each until reaching 24 m. The measurements for Tx and Rx combinations included two antenna polarization combinations, i.e., V–V and V–H. For the LOS, the bore sight alignments of the two antennas were used. Whereas, in the NLOS scenario, the received power was determined when the transmitting antenna was rotated to make sure there was an obstruction resulting in no clear optical path between the Tx and Rx antennas. At this point, there was no aligning bore sight between the two antennas. 

The complete description of the channel sounder, as well as the measurement setup, are provided. On the transmitting end, the Rhode and Schwarz SMB 100A radio signal generator was used to generate continuous wave (CW) signals, which then radiated across a wireless medium. This signal generator has a frequency range of 100 KHz to 40 GHz. A Rhode and Schwarz FSIQ 40 signal analyzer was used at the receiver end to receive the continuous wave signal from the SMB 100A signal generator. The frequency range of this FSIQ 40 signal analyzer is 20 to 40 GHz with a maximum bandwidth of 120 MHz. Two identical LB-180400-KF broadband horn antennas with frequency ranges of 18 to 40 GHz were used in this setup for transmitting and receiving radio signals. They had a nominal gain of 15 dBi, a low VSWR of 1.5:1, and a uniform gain across the frequency ranges, resulting in efficient performances and directionality. The antennas were also linearly polarized and could handle 10 W continuously and 20 W peak output. In the elevation plane, the half-power beam width (HPBW) had a minimum of 21 degrees and a maximum of 42 degrees, whereas, in the H-plane, it was a minimum of 17 degrees and a maximum of 45 degrees.

**Figure 3:**
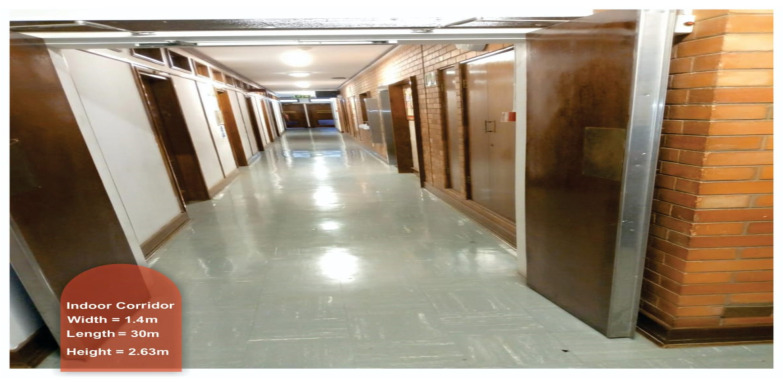
The indoor corridor environment.

**Figure 4:**
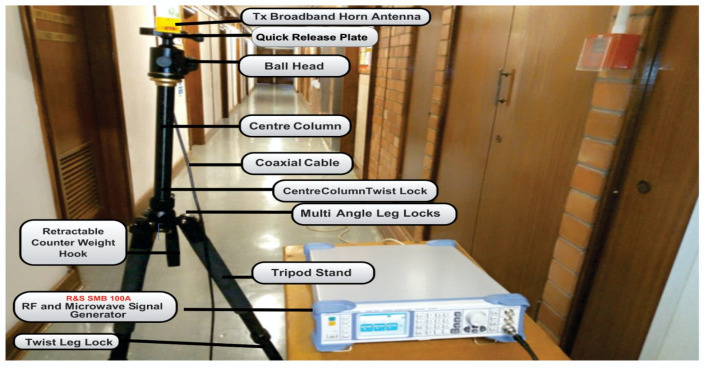
The transmitter setup.

**Figure 5:**
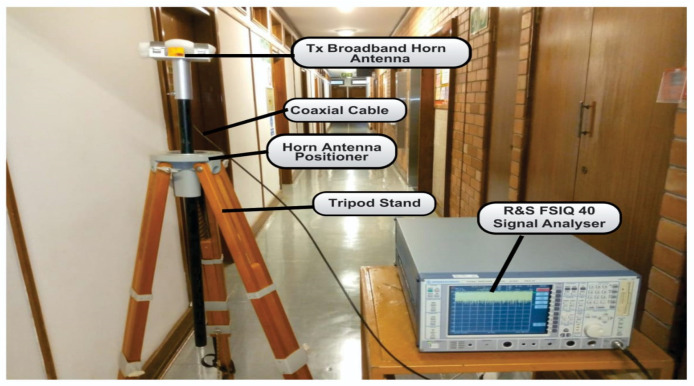
The receiver setup.

**Figure 6:**
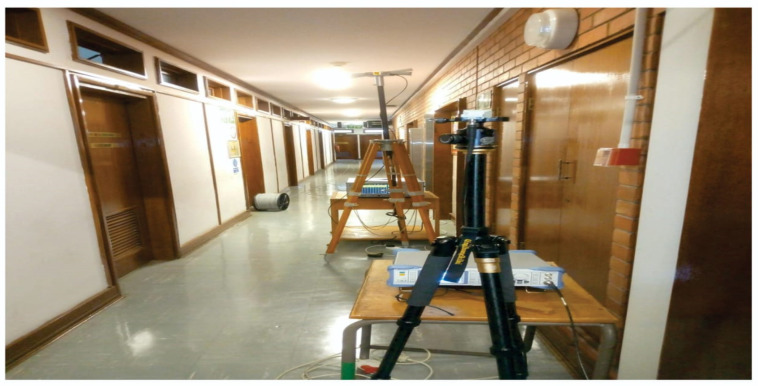
The setup of the transmitter and the receiver in the indoor corridor environment.

The measurements were conducted at the height of 1.6 m for the transmitting antenna and 2.3 m for the receiving antenna above the floor for both LOS and NLOS situations. Figure 3, Figure 4, Figure 5 and Figure 6 present the transmitter setup, receiver setup, and indoor corridor environment. Table 1 lists the channel sounder’s parameter settings. 

**Figure 7:**
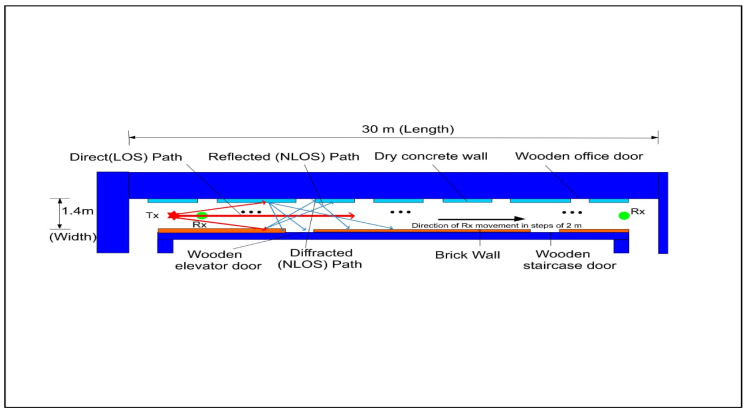
Floor plan of the indoor corridor environment [14].

The measuring campaigns took place on the 5th floor of the Electrical, Electronic, and Computer Engineering Department at the University of KwaZulu-Natal in Durban, South Africa. The length, height, and width of the indoor corridor are 30, 2.63, and 1.4 m, respectively. The corridor is made up of dry concrete brick walls, a square tiled floor, an elevator, and wooden office doors. The floor plan is shown in Figure 7. The indoor corridor was used for both the LOS and NLOS scenarios of the campaign.

The transmission of a continuous wave signal between the two broadband horn antennas used in the transmitting and receiving ends had antenna heights of 1.6 m at the transmitting antenna stand and 2.3 m at the receiver’s end. Both polarizations were placed in the vertical and horizontal directions; the center frequencies used were 28 and 38 GHz. For the V–V polarization, the Tx and Rx broadband horn antennas were vertically polarized. On the other hand, the Tx antenna for the V–H was vertically polarized, while the Rx antenna was horizontally polarized. The transmitting antenna was placed at one end of the corridor, and the receiver antenna was moved away from the transmitter by 2 m until it reached a distance of 24 m. Meanwhile, specialists in this field proposed a reference distance of 1 m between the transmitter and receiver. With a reference distance do = 1 m, the number of Tx–Rx separation distances was 13. When both antennas were aligned on the bore sight with no obstacles in the transmitting signal path between them, the LOS scenario was evaluated. However, in the NLOS, the received power was determined when the transmitting antenna was rotated to make sure that there was an obstruction resulting in no clear optical path between the Tx and Rx antennas. At this point, there was no aligning bore sight between the two antennas. The floor layout and the inside corridor are depicted in detail in Figure 3 and Figure 7, respectively.

It is worth noting that during the campaign, measuring cautions were observed in order to acquire accurate measurements. During the campaign, all doors were closed and human movement along the inside corridor was prohibited. In addition, all objects in the corridor were eliminated. The path loss was calculated using Equation (1), having considered the transmitted power $P_{t}$, received power $P_{r}$, gain of the transmitting antenna $G_{t}$ as well as the gain of the receiving antenna $G_{r}$. The path loss equation (all parameters in dB) is as follows, as computed in [21]:

(1) $$P_{L}=P_{t}-P_{r}+G_{r}+G_{t}$$

### 3.2. Path Loss Propagation Models

There is a general classification of models that requires minimal site or path details and that counts hindrances or obstructions as components of the distance-dependent losses, whereas site-specific models assess the losses due to each hindrance separately. These models are taken into account by placing the measured variables into a generic phrase. Approximately four major path loss propagation models are frequently used: two are single-frequency models, while the other two are multi-frequency models. Closed-in (CI), floating intercept (FI), CI model with a weighted frequency (CIF), and alpha–beta–gamma (ABG) are the acronyms [52,54,55,56,57,58,59,60,61,62,63,64,65,66,67,68].

Models in propagation path loss can be used to reflect the effects of path loss on the signal at the receiving end on a wide scale. It is a useful tool for calculating signal attenuation as it travels from the transmitter to the receiver, taking into account the propagation distance and other factors. The models differ in that some specify the topographical profile for easy signal analysis, while others just use the carrier frequency and distance to determine their targets [69,70]. The CI, CIF, and ABG (alpha–beta–gamma) path loss models are stochastic in nature, yet they capture the phenomenon of large-scale propagation across a given distance, keeping in mind that they can work at all appropriate frequencies in a given environment. The CI and CIF models are found to be equivalent to the standard forms of 3GPP path loss models (the FI and ABG models). Only the floating constant and the free space constant, which are dependent on propagation frequency and observance of the free space reference distance of 1 m [40,71,72,73,74,75], are relevant in this case.

#### 3.2.1. The Close-In (CI) Free Space Reference Distance Path Loss Prediction Model

This is a model that has its primary principle on the anchor point and depends on the frequency in the free space. The free space path loss (FSPL) is present in the model parameter, which also depends on the frequency of the carrier propagating signal (f in GHz). The distance between the transmitter and the receiver (d in meters), as well as the chosen reference distance ($d_{o}$), are also important factors. The CI model has one parameter to be calculated in dB, i.e., PLE (n) [42,75,76]. The equation for the model is shown in (2).

The reference distance of the CI model is 1 m, given by [40,75]:

(2) $$P_{L}^{CI}\;\left(d\right)\left[dB\right]=FSPL\left(f,d_{o}\right)\left[dB\right]+10.n.\log\left(\frac{d}{d_{o}}\right)+X_{\sigma SF}^{CI}$$

For *d* ≥ $d_{o}$, where $d_{o}=1m$

Where $X_{\sigma SF}^{CI}$ is a zero mean gaussian random variable with a standard deviation σ in *dB.*

(3) $$FSPLf,\;\left(d_{o}\right)\;\left[dB\right]=10log_{10}\left(\frac{4\pi d_{o}}{\lambda }\right)$$

To determine the CI path loss model, the PLE *n* is found using the MMSE approach, which commiserates with the data measured by adopting a physical anchor location that represents the free space power transmitted from the antenna at the transmitter out to the reference distance of the CI $d_{o}.$ The reference distance $d_{o}\;of\;1m$ is utilized in the mmWave CI model, which was proposed as a standard in [39] and [75]. Far-field radiation patterns (Fraunhofer distances) from high-gain directional antennas may be greater than 1 m from the antenna, but the CI path loss model may simply be converted to a 1 m reference distance by assuming that the far field begins at 1 m (even if it does not).

(4) $$FSPL\left(f,d_{o}\right)\left[dB\right]=10log_{10}{\left(\frac{4\pi fd_{o}}{c}\right)}^{2}\;$$

(5) $$FSPL\left(f,1m\right)\left[dB\right]=10log_{10}{\left(\frac{4\pi f}{c}\right)}^{2}$$

where c represents the speed of light.

From (1), the shadow-fading random variable is

(6) $$X_{\sigma SF}^{CI}=PL^{CI}\left(d\right)\left[dB\right]-FSPL\left(f,1m\right)\left[dB\right]-10.n.\log\left(d\right)$$

Let $A=PL^{CI}\left(d\right)\left[dB\right]-FSPL\left(f,1m\right)\left[dB\right]\;and$ D = 10.n.log(d)

Therefore,

(7) $$X_{\sigma SF}^{CI}=-nD\;$$

Then, the standard deviation of the random variable $X_{\sigma SF}^{CI}\;is$

(8) $$\sigma^{CI}=\sqrt{\sum^{\;}\frac{X_{\sigma }^{CI^{2}}}{N}}=\sqrt{\sum^{\;}\frac{{\left(A-nD\right)}^{2}}{N}}$$

where N represents the number of measured path loss points.

In order to obtain the PLE at the optimum value and $\sigma^{CI},$ the partial differential of the numerator of Equation (8), with respect to the value of PLE, is equal to zero. Then,

(9) $$\frac{\partial \left[\sum^{\;}{\left(A-nD\right)}^{2}\right]}{\partial n}=0\;$$

(10) $$\sum^{\;}2D\;\left(nD-A\right)=0$$

(11) $$2\sum^{\;}D\;\left(nD-A\right)=2\left(n\sum^{\;}D^{2}-\sum^{\;}DA\right)$$

Therefore, from Equation (11), $n=\frac{\sum^{\;}DA}{\sum^{\;}D^{2}}$

The minimum shadow fading (SF) standard deviation for the CI model is

(12) $$\sigma_{min}^{CI}=\sqrt{\frac{\sum^{\;}{\left(A-D\frac{\sum^{\;}DA}{\sum^{\;}D^{2}}\right)}^{2}}{N}}$$

The closed-form solution value was processed through MATLAB, *n*, and can be expressed using a Matrix formation;

(13) $$n=A^{T}{\left(D^{T}D\right)}^{-1}D$$

(14) $$\sigma_{min}^{CI}=\;\sqrt{\frac{\sum^{\;}{\left(A-A^{T}{\left(D^{T}D\right)}^{-1}D\right)}^{2}}{N}}$$

#### 3.2.2. The Floating Intercept (FI) Free Space Reference Distance Path Loss Prediction Model

This model relies on two major integral parts, i.e., the line slope, as well as the floating intercept. The FI model is good at achieving the optimal minimum error fit for the path loss values, [39,75]. It can be expressed by Equation (15);

The equation of this path loss model is [39]:

(15) $$PL^{FI}\left(d\right)\left[dB\right]=\propto +10.\beta log_{10}\left(d\right)+X_{\sigma SF}^{FI}$$

This model adopts ∝ as the floating intercept in dB, and the slope of the line is β (not as in PLE).

Moreover, ∝ and β were the two parameters adopted by this FI model to make it different from the CI model. The zero-Gaussian shadow fading (in dB) variable over the mean path loss on a specified distance was $X_{\sigma SF}^{FI}.$ The best fit, such as the CI model, entailed solving ∝ and β and minimizing σ; the closed-form optimized solutions are provided below. It is worth noting that the FI model requires two model parameters, whereas the CI model only requires one.

Let $B=PL^{FI}\left(d\right)\left[dB\right]$, and $D=10.log_{10}\left(d\right)$

Then, Equation (15) becomes,

(16) $$B=\propto +\beta D+X_{\sigma SF}^{FI}.$$

(17) $$X_{\sigma SF}^{FI}=B-\propto -\beta D$$

Then the standard deviation of the random variable is $X_{\sigma }^{FI}$

(18) $$\sigma^{FI}=\sqrt{\frac{\sum^{\;}X_{\sigma }^{FI^{2}}}{N}}=\sqrt{\frac{\sum^{\;}{\left(B-\propto -\beta D\right)}^{2}}{N}}$$

If we want to minimize $\sum^{\;}{\left(B-\propto -\beta D\right)}^{2},$

It implies that its partial derivate should be zero with respect to β and ∝; that is,

(19) $$\frac{\partial \left[\sum^{\;}{\left(B-\propto -BD\right)}^{2}\right]}{\partial \propto }=\sum^{\;}2\left(\propto +\beta D-B\right)\;$$

(20) $$2\left(N\propto +\beta \sum^{\;}D-\sum^{\;}B\right)=0\;$$

(21) $$N\propto +\beta \sum^{\;}D-\sum^{\;}B=0\;$$

(22) $$\frac{\partial \left[\sum^{\;}{\left(B-\propto -BD\right)}^{2}\right]}{\partial \beta }=\sum^{\;}2D\left(\propto +\beta D-B\right)\;$$

(23) $$2\left(\propto \sum^{\;}D+\beta \sum^{\;}D^{2}-\sum^{\;}DB\right)=0\;$$

(24) $$\propto \sum^{\;}D+\beta \sum^{\;}D^{2}-\sum^{\;}DB=0\;$$

Now, combining Equations (21) and (24),

(25) $$\propto =\frac{\sum^{\;}D\;\sum^{\;}DB-\sum^{\;}D^{2\;}\sum^{\;}B}{{\left(\sum^{\;}D\right)}^{2}-N\sum^{\;}D^{2}}$$

(26) $$\beta =\frac{\sum^{\;}D\;\sum^{\;}B-N\sum^{\;}DB}{{\left(\sum^{\;}D\right)}^{2}-N\sum^{\;}D^{2}}$$

The minimum shadow-fading standard deviation can be obtained by substituting ∝ and β in Equation (18), with (25) and (26), respectively [39]:

Then, the minimum SF standard deviation is now;

(27) $$\beta ={\left(D-\bar{D}\right)}^{T}\;{\left(D-\bar{D}\right)}^{T}\;{\left(D-\bar{D}\right)}^{-1}\;\left(B-\bar{B}\right)$$

(28) $$\propto =\left(B-\bar{\beta D}\right)$$

Column vectors B and D have mean values of $\bar{B}$ and $\bar{D}$, respectively.

## 4. Results and Discussion

This section details the discussion of results in the LOS and NLOS analyses of path loss models as well as the performance assessments of the model’s variables. The Rohde & Schwarz FSIQ 40 signal analyzer was used to capture the LOS and NLOS data, which were coupled to a broadband horn antenna. The data were analyzed (using MATLAB) in order to propose viable large-scale path loss prediction models using the minimum mean square error (MMSE) method, which fits the measured data in an indoor corridor environment at 28 and 38 GHz frequencies in two different antenna polarizations.

### 4.1. LOS Measurement Evaluation Study Results and Discussion

**Figure 8:**
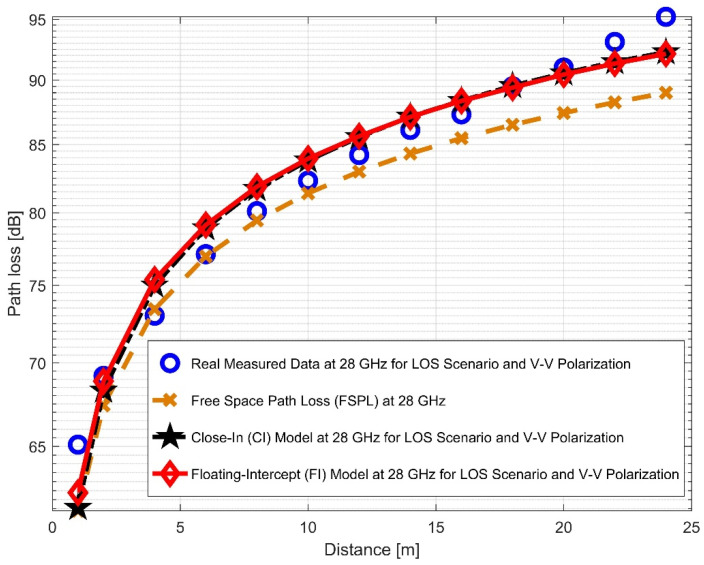
Path loss versus distance at 28 GHz for the LOS scenario and V–V polarization.

**Figure 9:**
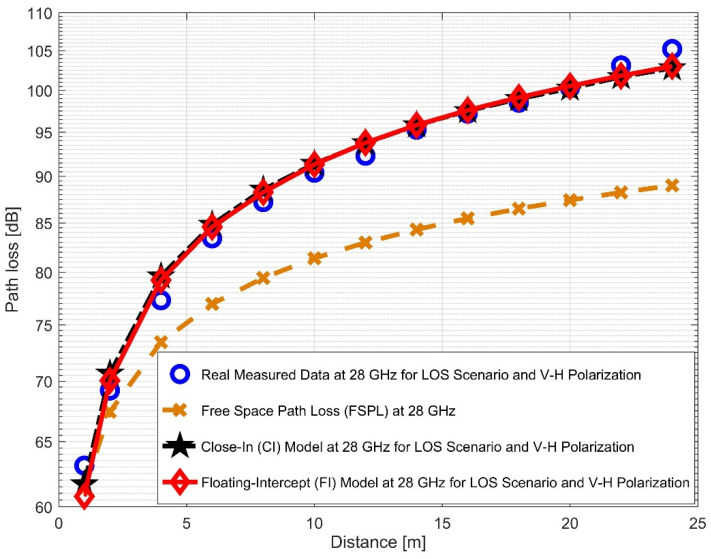
Path loss versus distance at 28 GHz for the LOS scenario and V–H polarization.

This experiment examined two frequency bands (28 and 38 GHz) at two antenna polarizations, revealing the findings and allowing for a comparison with the other propagation models. Figure 8 and Figure 9 present the measured data and FPSL curves, illustrating the CI and FI models for the 28 GHz frequencies at vertical-to-vertical (V–V) polarization and vertical-to-horizontal (V–H) polarization, respectively. The CI and FI path loss model curves overlapped with each other and accurately fit the real measured data in both antenna polarizations. They still closely matched the V–H polarization than the V–V. When the experiment was run using 28 GHz V–V antenna polarization, the PLE value was 2.254. However, when the experiment was run using a V–H antenna polarization, the PLE value increased to 2.979, indicating that the signal degraded more in the V–H polarization than in the V–V mode. Moreover, the values of α for the V–V and V–H were 58.8294 and 59.9354, respectively. Nevertheless, the value of *β*^FI^ rose from 2.154 to 3.05. It was observed that the value of $\sigma^{FI}$ declined from 1.7431 (for V–V) to 1.3008 (for V–H).

**Figure 10:**
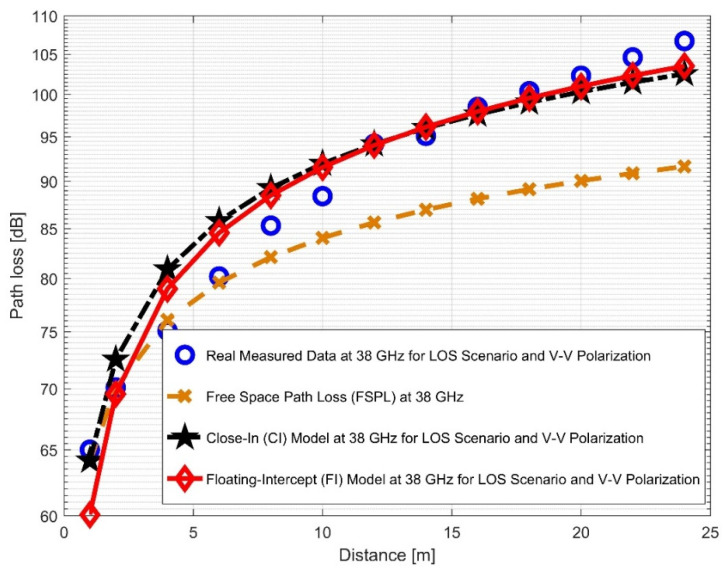
Path loss versus distance at 38 GHz for the LOS scenario and V–V polarization.

**Figure 11:**
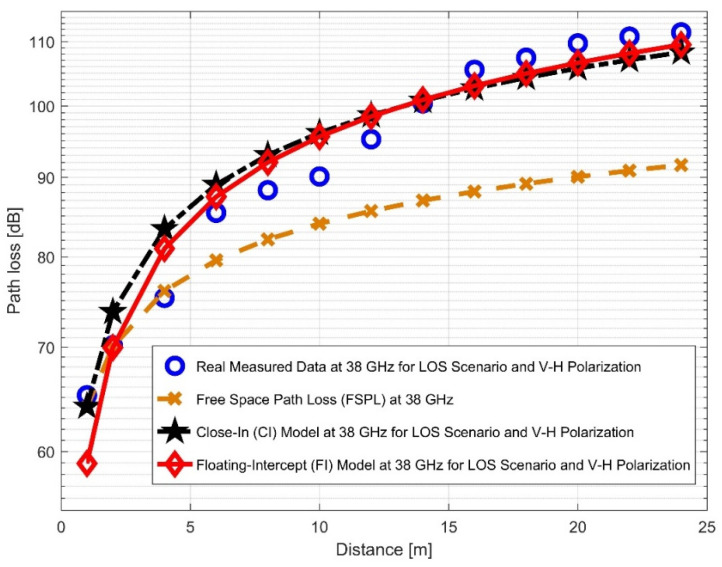
Path loss versus distance at 38 GHz for the LOS scenario and V–H polarization.

When transitioning from the V–V to the V–H antenna polarizations in the 38 GHz frequency band, there was an increase of 0.4186 in the path loss exponent. This shows that while changing antenna polarizations at a 38 GHz frequency, signal degradation occurred. The curves are shown in Figure 10 and Figure 11. When contrast to the 28 GHz frequency, the PLEs for both antenna polarizations were significantly larger at 38 GHz. This is because increased scattering and penetration losses were generated by an increase in the signal attenuations at higher frequencies. The various irregular materials, such as bricks, concrete, and other building materials (e.g., wooden doors and elevator doors), produce interference and may potentially impact the outcome. The standard deviation for shadow fading $\sigma^{CI}$ for the V–V antenna polarization was 3.1874; nevertheless, it climbed slightly to 4.1001 for the V–H antenna polarization. This demonstrates that there are many signal fluctuations in both situations. Additionally, $\alpha^{FI}$ has a 1.6439 increase from the V–V to the V–H. While $\beta^{FI}$ goes from 3.1461 to 3.6625, the $\sigma^{FI}$ min value rises from V–V to V–H by 0.8016.

**Figure 12:**
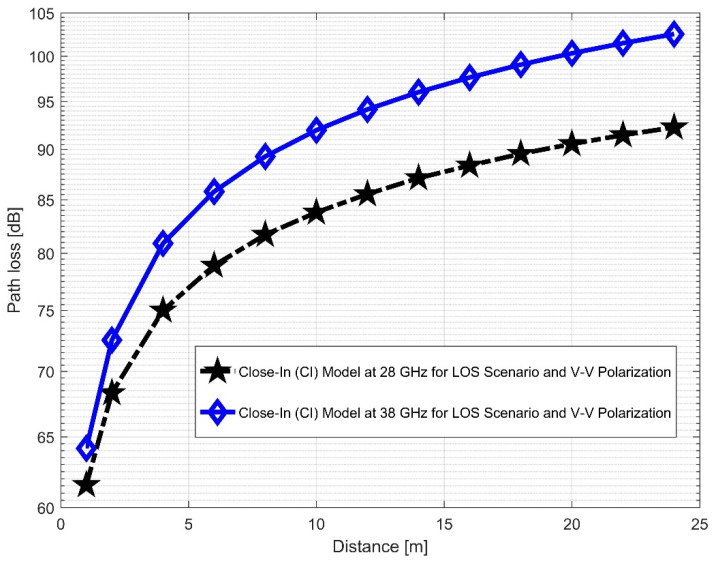
CI path loss versus distance at 28 and 38 GHz for the LOS scenario and V–V polarization.

**Figure 13:**
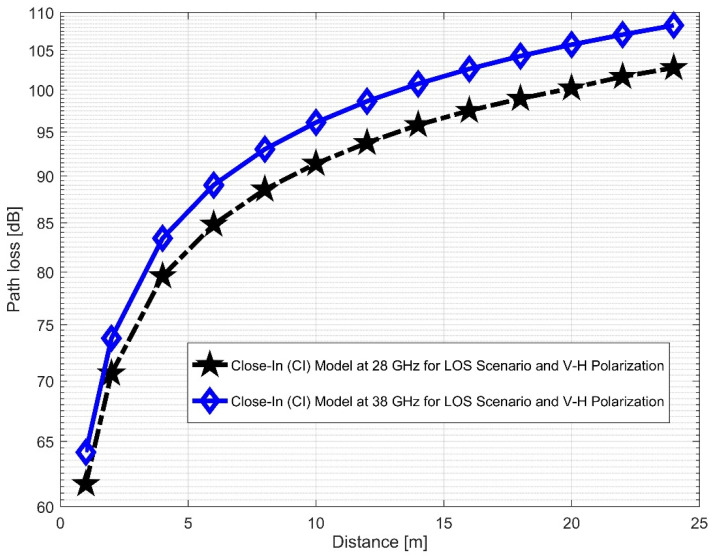
CI path loss versus distance at 28 and 38 GHz for the LOS scenario and V–H polarization.

**Figure 14:**
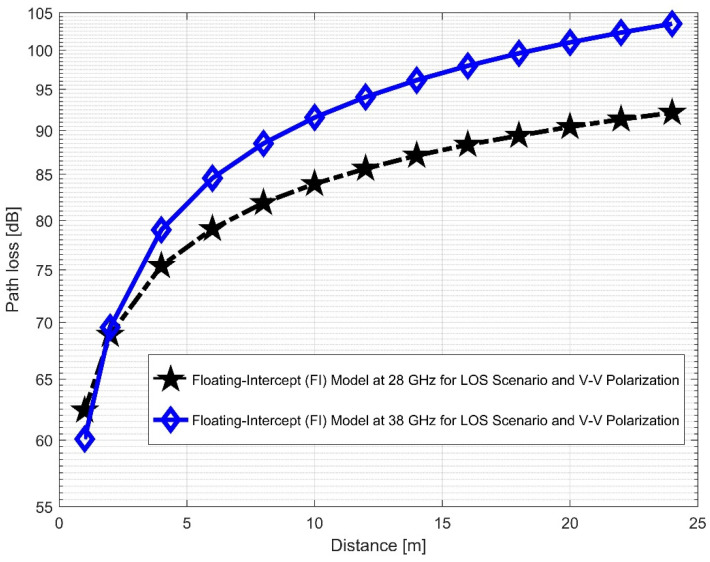
FI path loss versus distance at 28 and 38 GHz for the LOS scenario and V–V polarization.

**Figure 15:**
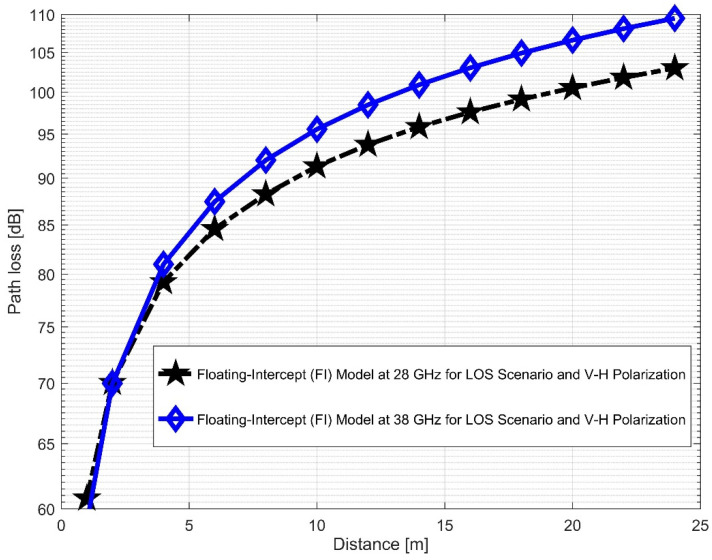
FI path loss versus distance at 28 and 38 GHz for the LOS scenario and V–H polarization.

Figure 12 and Figure 13 compare the LOS CI at 28 and 38 GHz for the two antenna polarizations considered. It was discovered that path loss was more pronounced at 38 GHz than at 28 GHz for the V–V polarization. This indicates that higher frequencies are subject to the numerous propagation effects and path losses. The correlations between the path loss values in FI (in Figure 14 and Figure 15) are very close to V–H, but have larger differences in the V–V polarization (similar to the CI case). This means that in the LOS scenario, both CI and FI behave similarly. Table 2 presents the LOS parameters of the CI and FI path loss models.

### 4.2. NLOS Measurement Evaluation: Study Results and Discussion

**Figure 16:**
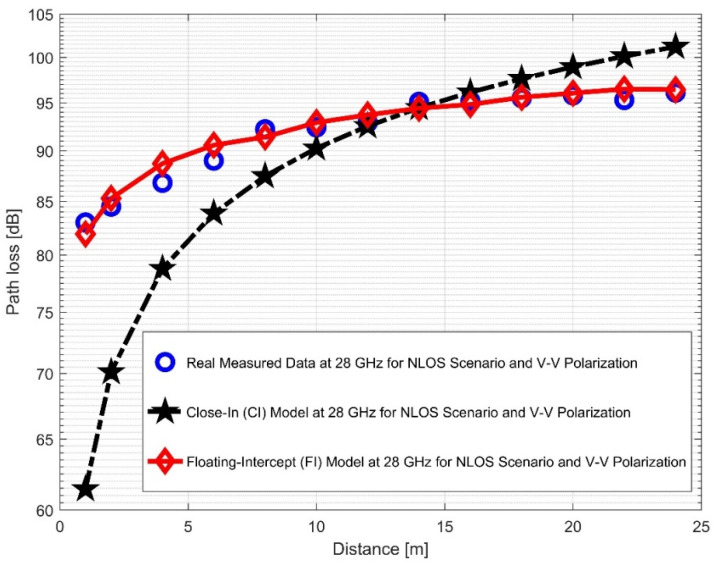
Path loss versus distance at 28 GHz for the NLOS scenario and V–V polarization.

**Figure 17:**
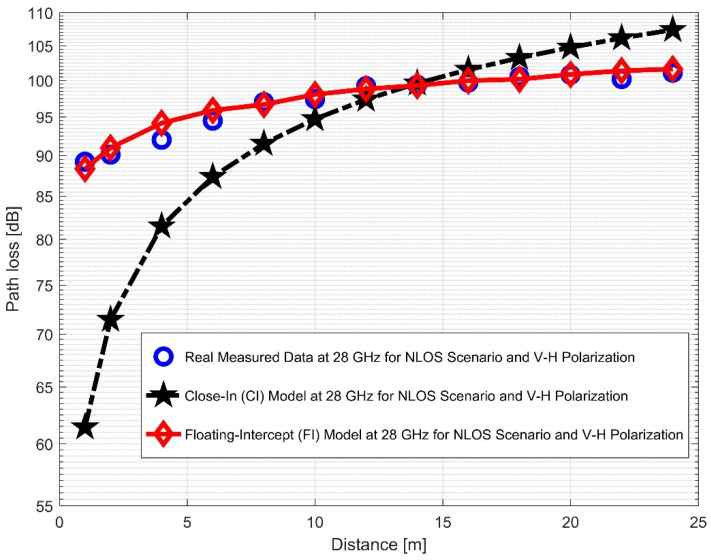
Path loss versus distance at 28 GHz for the NLOS scenario and V–H polarization.

The CI and FI models for both vertical-to-vertical (V–V) and vertical-to-horizontal (V–H) antenna polarizations were evaluated using NLOS measurements. It is clear that, compared to the 28 GHz frequency band, the reflection from the corridor’s walls—made of dry concrete and bricks—is more difficult when the values of the PLE in both the V–V and V–H are maximum at 38 GHz. Plots for the measured values, CI, FI, and FSPL models for the V–V and V–H in the 38 GHz and 28 GHz frequency bands are shown in Figure 16 and Figure 17, respectively.

The PLEs of the 28 GHz in V–V and V–H antenna polarizations differ, with the PLE for the V–H polarization increasing by about 0.445. The standard deviation of 0.78 decibels was approximately the same for both polarizations. The PLE in the 38 GHz situation was 2.8207 in the V–V polarization, increased by 0.6475 in the V–H polarization, and a notable increase was observed in the value of the standard deviation, which rose from 1.6283 to 2.9396 dB. The changes in the PLE and standard deviation were caused by changes in the antenna polarization due to different wave guiding effects, diffraction, and reflections at the two antenna polarizations, culminating in constructive interference of the multiple signal components that reached the receiver in both cases.

**Figure 18:**
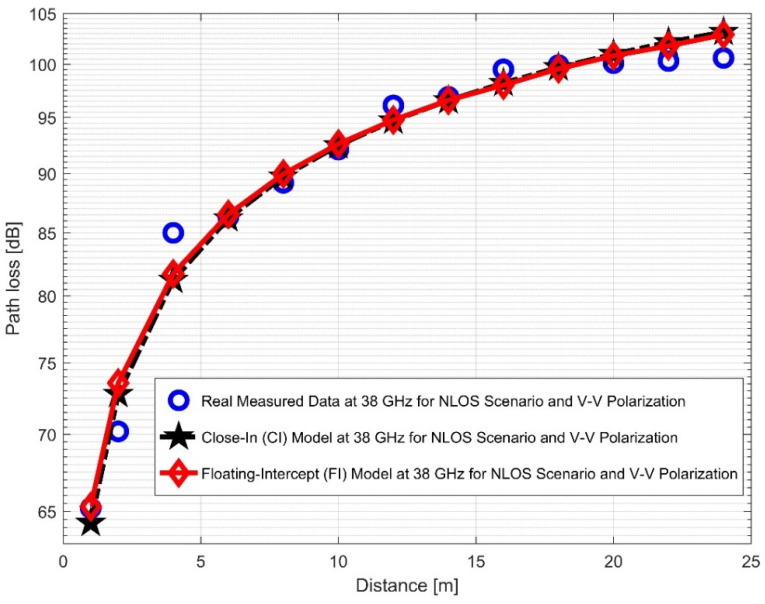
Path loss versus distance at 38 GHz for the NLOS scenario and V–V polarization.

**Figure 19:**
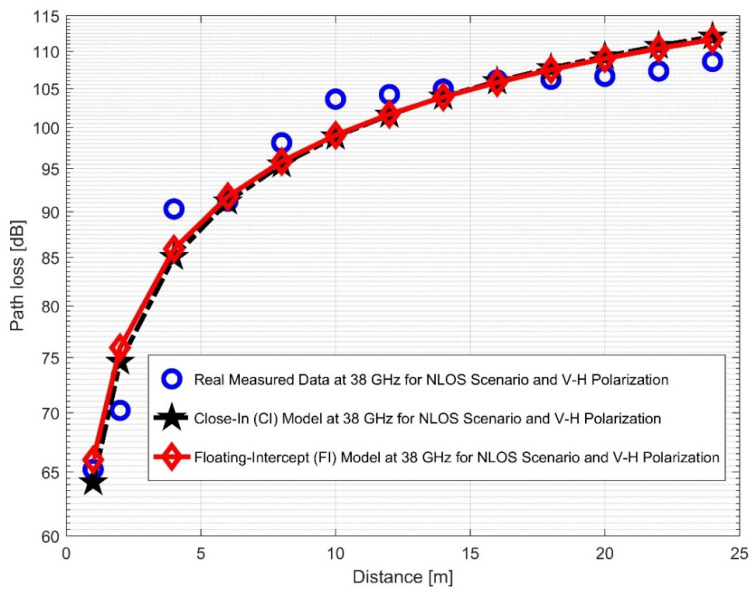
Path loss versus distance at 38 GHz for the NLOS scenario and V–H polarization.

When considering the 38 GHz frequency, the higher value of PLE and standard deviation indicated that there was a low wavelength and a higher path loss at a higher frequency. Figure 18 and Figure 19 display the plots for both V–V and V–H antenna polarizations. The floating intercept values (β) at 28 GHz were 1.0558 and 0.9722, which were approximately the same in V–V and V–H. However, at 38 GHz, the floating intercept value rose to 2.7254 in V–V polarization and 3.3064 in V–H polarization.

**Figure 20:**
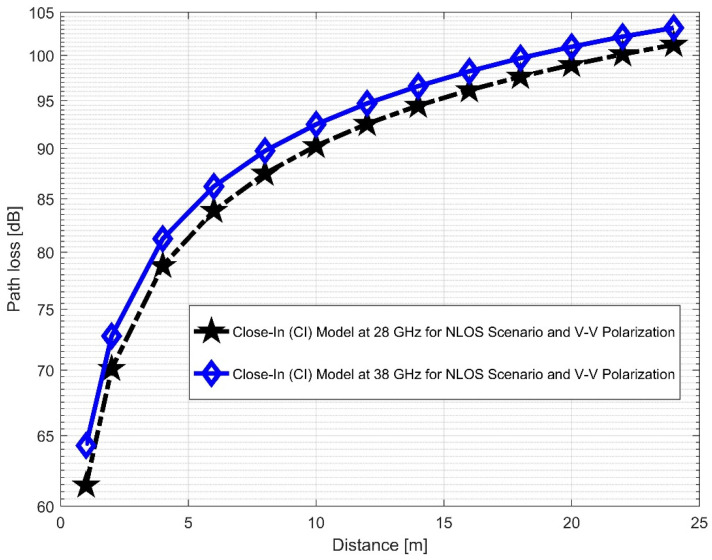
CI path loss versus distance at 28 and 38 GHz for the NLOS scenario and V–V polarization.

**Figure 21:**
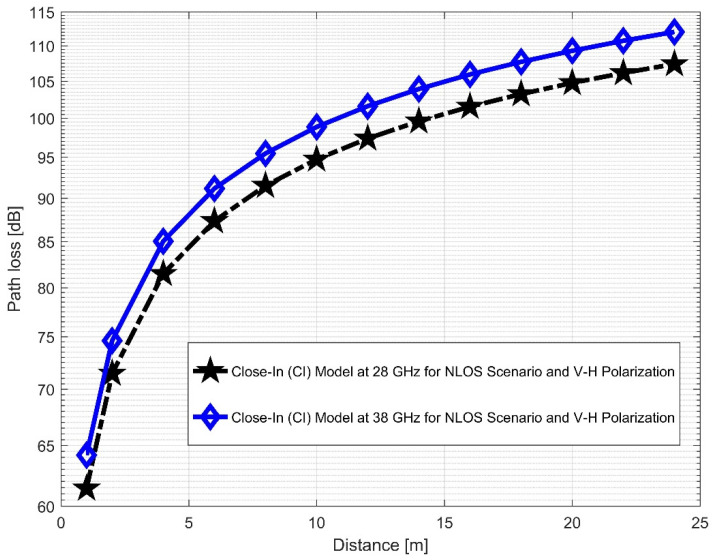
CI path loss versus distance at 28 and 38 GHz for the NLOS scenario and V–H polarization.

**Figure 22:**
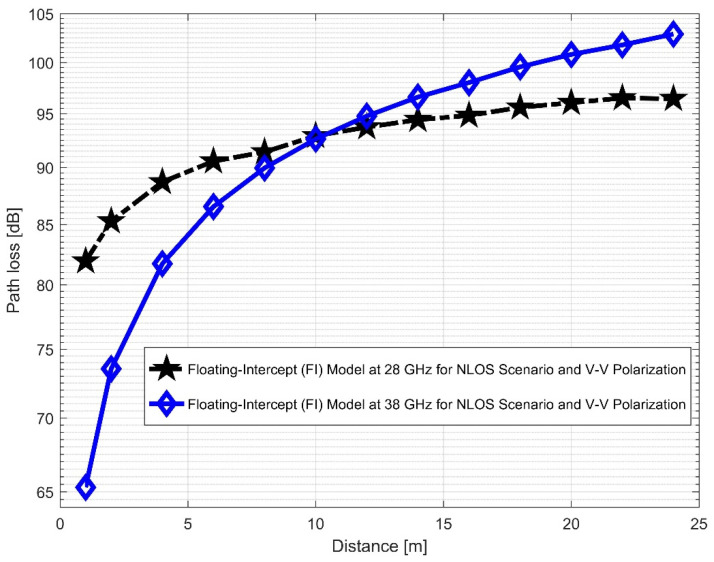
FI path loss versus distance at 28 and 38 GHz for the NLOS scenario and V–V polarization.

**Figure 23:**
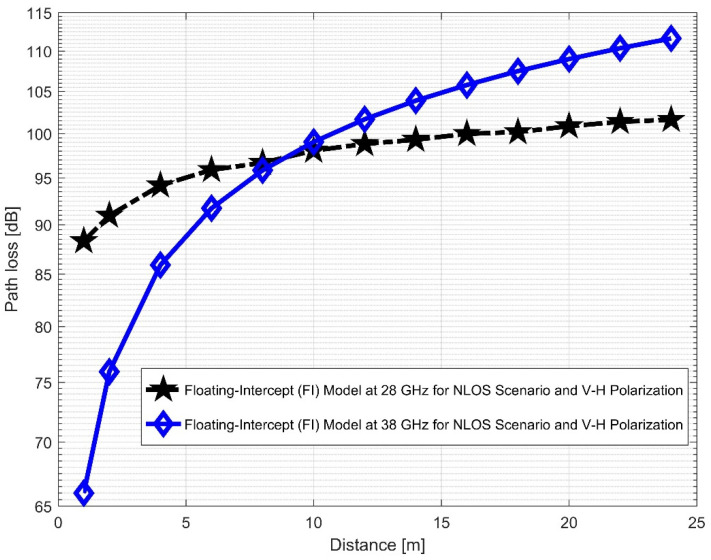
FI path loss versus distance at 28 and 38 GHz for the NLOS scenario and V–H polarization.

Furthermore, the values of $\sigma_{min}^{CI}$ in the 28 GHz frequency band had a sharp increase in both polarizations when compared to the value in the LOS scenario. The value was 8.1287 for (V–V) and 10.4790 for (V–H), while the 38 GHz values increased slightly from 1.6822 (V–V) to 3.0257 (V–H). This indicates that the signal performance in the NLOS was better in the 38 GHz frequency band when compared to the 28 GHz in both polarizations (although the contrast occurred for the LOS situations). The rate of increase in penetration losses at 28 GHz was higher in the NLOS than 38 GHz (a similar behavior occurred in the experimental analysis by [69,77]). This behavior is seen in the plotting of the path losses shown in Figure 16 and Figure 17 for 28 GHz, and in Figure 18 and Figure 19 for 38 GHz. The behaviors of the curves (of CI path losses for 28 and 38 GHz in the NLOS for V–V antenna polarization) in Figure 20 were strikingly similar. This demonstrates that the path loss follows nearly the same patterns at both frequencies, albeit with a higher value at 38 GHz. However, in the case of V–H polarizations, as seen in Figure 21, it almost follows the same pattern as the V–V situation, but with a slight difference in the value of the path loss in the frequency bands. This still justifies the fact that the path loss will always be greater at higher frequencies. Figure 20 demonstrates improved sensitivity. The path loss in the 38 GHz band was higher in the FI cases of Figure 22, as expected. However, there was a case where the value was similar when the distance between the Tx and the Rx was 10 m. Figure 23 depicts the same situation, but with a higher path loss value for the two frequencies. At a distance of 10 m, the path loss values also coincided. Table 3 and Table 4 display the parameters of the CI and FI path loss models for both polarizations for the NLOS and LOS, respectively. Figure 20, Figure 21, Figure 22 and Figure 23 show comparisons of the CI and FI plots for the 28 and 38 GHz frequency bands.

Taking a look at the overall measurement results in the LOS and NLOS, both models clearly fit the measured path losses and produce comparable results in both antenna polarizations. The PLE was also much higher in the V–H polarization of the NLOS scenarios due to signal degradation along the path from Tx to Rx. At 38 GHz, the highest value was 3.33 (V–H). This demonstrates that reflections from dry concrete and bricks become more difficult at this frequency when compared to other frequencies. This was due to the lack of direct bore sight between the Tx and Rx antennas. The Rx (as it transmits through the path from the Tx antenna) only relies on signal diffractions and reflections from obstacles. Furthermore, the values of $\sigma_{min}^{CI}$ and $\alpha_{FI}$ increase to a peak at 28 GHz, rather than 38 GHz, in the NLOS scenario. This demonstrates that both antenna polarizations have better signal performances at 38 GHz. The slope of the path loss curve was roughly the same as the PLE values in both LOS and NLOS situations. This means they behaved similar in this environment. In the NLOS scenario, the minimum standard deviation values were much higher. This is due to the richness of the reflections and diffractions, which allowed for constructive inference of the multiple signal components reached on the Rx side, particularly in the 38 GHz V–V polarization. The NLOS scenario’s general behavior, which was characterized by a higher path loss, was caused by the diffraction effect, which disrupted signal transmissions. Because the Tx and Rx are not in the bore sight, obstructions between them tend to interfere with the signal transmission. Some obstacles reflect signals at specific frequencies, while others simply absorb and garble them. However, in either case, they degrade the signals, especially when the power budget is limited. The electrical properties of the material causing the obstruction also have significant impacts on the signal. Some are excellent conductors, while others are excellent insulators.

### 4.3. 5G Wireless Network Comparison of the Obtained Propagation Parameters with Other Indoor mmWave Outcomes

The propagation variable values are listed in a range in Table 5 (lower—upper). This is due to the fact that the work was conducted under a variety of LOS and NLOS conditions. Due to the accumulation of many path components, the lower range of PLE in this work under the LOS scenario was 2.23 at 28 GHz V–V. In the 73 GHz frequency range for V–H antenna polarization, the authors of [39] found the greatest PLE for an indoor environment with the measuring campaign conducted in a sizable open hall. The maximum upper value of PLE (3.47) in this investigation, however, was recorded at 38 GHz for V–H polarization. Practically all of the PLE values for the LOS in this study had FSPL exponents higher than 2. This demonstrated that there were few hints of depolarization at 28 GHz for V–V, 28 GHz for V–H, 38 GHz V–V, and 38 GHz V–H.

In the FI model, the frequency-dependent **α**^FI^ model had a value of 58.83 dB (V–V) that varied slightly with the free space path loss (FSPL) at 1 m for 28 GHz, which was 61.4 dB. In the V–H, however, the value was 59.94. At 1 m, the FSPL at 38 GHz was 64 dB. However, the V–V value obtained in this study was 60.54 and the V–H value was 62.19. The lowest range in the literature [46] was at a frequency of 6.5 GHz (in an indoor corridor environment, with a value of 40.7) and the highest was at 73 GHz, with a value of 101.1, as recorded in [39]. At 4.5 GHz for V–V polarization, the slope value ***β***^FI^ had the lowest value of 2.16; however, the lowest value of our experiment was 2.16 for 28 GHz V–V polarization. The value of ***σ***^CI^ and ***σ***^FI min^ in both the lower and higher ranges was also consistent with the mean values in all of the literature studies taken into consideration. The highest value of ***β***^FI^ was recorded at [77] for a frequency of 28 GHz.

The lowest range of the PLE value, which was 1.97 at 4.5 GHz frequency V–V [51], was lower in the NLOS scenario than our 2.384 estimate. Our PLE’s greatest value, 3.47, was less than the number in the highest range, 73 GHz [39]. These findings demonstrate that our study remains consistent with previous studies in the literature. The rise in signal degradation, particularly with a higher frequency, is a clear NLOS indicator. Depolarization occurs as a result of this. The range value for the **α**^FI^ model ranges from 16.22 at 4.5 GHz to 120.5 at 73 GHz [39], with 16.22 being the lowest number and 120.5 being the highest. Our work’s lowest value of **α**^FI^ is 65.11 at 38 GHz for V–V polarization, but it rises to 87.81 at 28 GHz for V–H polarization, which is still within the acceptable range for the works we took into consideration. The values in our work, however, fell within the range of the value ***β***^FI^ (documented in both upper ranges) [50]. The values of additional parameters, such as **σ**^CI^ and **σ**^FI min^, were congruent with those reported in the literature, after carefully contrasting the path loss parameters from the work with those from the literature. The majority of the work’s ranges were consistent; however, a key result was that these parameters differed based on the indoor environment. This is because different indoor settings in the literature were constructed from various materials, which caused the multipath components to vary and, thus, impacted the received signals. Different building materials and obstacles have varying penetration losses, dispersions, and fading. It is possible that some situations could experience signal attenuation because of air concentration, which can also be the reason for the various path loss propagation parameters.

The comparison results show that almost all of the path loss parameters in our work were within the range of the works considered in the literature. The main accomplishment was the better analysis of our work, which resulted in superior results in the PLE and standard deviation values. These two factors are critical when assisting engineers and researchers in budget calculations in 5G wireless network propagations in indoor environments. The study’s main goal was to examine single-frequency path loss models in an indoor environment at frequencies of 28 and 38 GHz. A thorough comparison of the path loss parameters was also performed to compare the values of the work’s parameters with those in the literature. Although the ranges were consistent with the majority of the work, one major finding was that these parameters varied depending on the indoor environment. This is due to the fact that various indoor environments in the literature were made of different materials, causing the multipath components to differ and, thus, affecting the received signals. Each obstacle and building material had different penetration losses, scattering, and fading. However, more measurements in these frequency bands will be required in the future, taking into account the losses of building materials and obstructions in each indoor environment. This will aid in the conception of a building structure-specific model.

## 5. Conclusions

This paper presented the results of an analysis of large-scale path loss models in an indoor corridor environment at 28 and 38 GHz frequency bands with two different antenna polarizations, in both LOS and NLOS scenarios. For all of the frequency bands investigated, measurements were taken using a channel sounder based on a Rohde & Schwarz SMB 100A radio frequency signal generator as the transmitter and a Rohde & Schwarz FSIQ 40 signal analyzer as the receiver. In this environment, two vertically polarized high-gain directional broadband horn antennas (for both vertical-to-vertical and vertical-to-horizontal polarizations_ were adopted, analyzed, and contrasted for LOS and NLOS communication scenarios. The single-frequency CI and single-frequency FI path loss models were used in this investigation. The measurement evaluations in an indoor corridor environment at 28 and 38 GHz frequency bands were the focus of this work. 

The LOS study results showed that when changing the antenna polarization from V–V to V–H at 28 GHz, the path loss values increased, but only slightly at 38 GHz, indicating that signal degradation was not too noticeable when changing the antenna polarization at 38 GHz. However, in the NLOS scenario, the PLE was higher at 38 GHz when compared to 28 GHz for both antenna polarizations. This was due to greater dispersion and penetration losses at higher frequencies. The minimum standard deviation values for CI and FI were quite near to one another for 28 GHz at both antenna polarizations. However, the minimum standard deviation for the 38 GHz frequency increased from 2.7 in the V–V polarization to 3.59 in the V–H polarization. The V–V antenna polarizations had better accuracy and path loss efficiencies than the V–H polarizations in both scenarios and frequencies, according to the results in both antenna polarizations. The proximity of the walls, the materials used for the walls, the floor, and other irregular items, such as wooden doors, concretes, and elevator doors along the hallway, all have an impact on how radio waves propagate indoors. Both of the models (CI and FI) employed had good overall performances. In order to achieve a better outcome, it will be a good idea to develop an improved CI–FI model. Moreover, the high reflection rate of power and penetration losses in millimeter wave propagation will require researchers to devise path loss models that are unique to each building’s structure in the future. 

## Figures and Tables

**Table 1:** Parameter specifications of the channel sounder.

Parameters	Configuration	Unit
Center frequency	28, 38	GHz
Bandwidth	100	MHz
Transmission signal	Continuous wave	
Tx and Rx Antennas	Broadband horn antenna	
Tx Antenna power	10	dBm
Tx Antenna height	1.6	m
Rx Antenna height	2.3	m
Tx and Rx antenna gain at 28 GHz	15	dBi
Tx and Rx antenna gain at 38 GHz	17	dBi
Tx and Rx antenna polarization	Vertical/horizontal	
Antenna size L × W × H	71 × 32 × 28.6	mm
Antenna weight	0.08	Kg

**Table 2:** The LOS comparative study results at 28 and 38 GHz frequencies.

28 GHz LOS communication scenario		
	V–V polarization	V–H polarization
PLE (n)	2.2254	2.9790
$\sigma_{min}^{CI}$ [dB]	1.7718	1.3425
$\alpha_{FI}$ [dB]	58.8294	59.9354
$\beta_{FI}$	2.1537	3.0540
$\sigma_{min}^{FI}$ [dB]	1.7431	1.3008
38 GHz LOS communication scenario		
	V–V polarization	V–H polarization
PLE (n)	2.7801	3.1978
$\sigma_{min}^{CI}$ [dB]	3.1874	4.1001
$\alpha_{FI}$ [dB]	60.5444	62.1883
$\beta_{FI}$	3.1461	3.6625
$\sigma_{min}^{FI}$ [dB]	2.7439	3.5455

**Table 3:** The NLOS comparative study results in 28 and 38 GHz frequencies.

28 GHz NLOS communication scenario		
	V–V polarization	V–H polarization
PLE (n)	2.8815	3.3303
$\sigma_{min}^{CI}$ [dB]	8.1287	10.4790
$\alpha_{FI}$ [dB]	81.8470	87.8146
$\beta_{FI}$	1.0558	0.9722
$\sigma_{min}^{FI}$ [dB]	0.7872	0.7796
38 GHz NLOS communication scenario		
	V–V polarization	V–H polarization
PLE (n)	2.8207	3.4682
$\sigma_{min}^{CI}$ [dB]	1.6822	3.0257
$\alpha_{FI}$ [dB]	65.1057	65.8500
$\beta_{FI}$	2.7254	3.3064
$\sigma_{min}^{FI}$ [dB]	1.6283	2.9396

**Table 4:** Single-frequency CI and FI path loss model parameters for all measured frequencies in the indoor channels.

LOS						
Frequency (GHz)	Polarization	CI		FI		
		*PLE (n)*	σ^CI^ [dB]	α^FI^ [dB]	*β* ^FI^	σ^FI min^ [dB]
28	V–V	2.2254	1.7718	58.8294	2.1537	1.7431
	V–H	2.9790	1.3425	59.9354	3.0540	1.3008
38	V–V	2.7801	3.1874	60.5444	3.1461	2.7439
	V–H	3.1978	4.1001	62.1883	3.6625	3.5455
NLOS						
28	V–V	2.8815	8.1287	81.8470	1.0558	0.7872
	V–H	3.3303	10.4790	87.8146	0.9722	0.7796
38	V–V	2.8207	1.6822	65.1057	2.7254	1.6283
	V–H	3.4682	3.0257	65.8500	3.3064	2.9396

**Table 5:** Comparison of indoor channels at mmWave frequency ranges for single-frequency path loss model parameters.

Ref.	Dist. (m)	Freq. Range (GHz)	Pol.	Envi.	Dimen. (m)	Materials Make	Sce.	*PLE (n)*	σ^CI^ [dB]	α^FI^ [dB]	*β* ^FI^	σ^FImin^ [dB]
[39]	4.1–21.3	28–73	V–V V–H	Indoor office	39.3 × 2.7 × 45.9	Cubicle partitions, doors, dry walls, elevator	LOS	3.5	1.8–8.6	60.4–101.1	0.5–1.6	1.6–15.8
[46]	1–40	6.5–38	V–V V–H	Indoor corridor	2.8 × 2.4 × 40	Plywood, glass doors, concrete walls, glass, and gypsum board.	LOS	0.6–1.9	1.31–2.8	40.7–70	0.9–1.4	1.12–3.1
[50]	1–22.7	4.5–38	V–V V–H	Indoor office	21 x 2.7 × 30	Concrete walls and gypsum board	LOS	1.13–3.87	2.18–5.6	41.45–83.79	0.33–1.77	1.12–3.97
[68]	1–50	26–39	V–V	Indoor office	2.41 × 2.89 × 40	Wooden doors, light concrete walls, a false ceiling of gypsum	LOS	1.14–1.53	4.25–4.94	62.12–65.86	1.03–1.61	4.24–4.94
[71]	4.1–21.3	28–73.5	V–V	Indoor office	39.3 × 2.7 × 45.9	Desks, chairs, cubicle partitions, doors, dry walls, elevator	LOS	1.1–1.3	1.8–2.4	60.4–77.9	0.5–1.2	1.8–2.3
[77]	14–50	26–38	V–V	Indoor Library	14 × 7 x 52	Non-tinted glass and printed hardboard frames	LOS	1.96–3.24	3.78–6.30	52.63–100.71	0.95–4.18	3.99–7.86
This Work	2–24	28–38	V–V V–H	Indoor Corridor	2.63 × 1.4 × 30	Dry concretes and bricks, staircase, elevator, and office wooden doors	LOS	2.23–3.47	1.34–4.10	58.82–62.19	2.15–3.66	1.30–3.55
[39]	4.1–21.3	28–73	V–V V–H	Indoor office	39.3 × 2.7 × 45.9	Cubicle partitions, doors, dry walls, elevator	NLOS	4.4- 6.4	10.9–15.9	81.6–120.5	1.3–2.6	8.0–11.3
[50]	1–22.7	4.5–38	V–V V–H	Indoor office	21 x 2.7 × 30	Concrete walls and gypsum board	NLOS	1.97–5.28	2.62–6.0	16.22–87.91	2.4–6.12	2.57–4.5
[71]	4.1–21.3	28–73.5	V–V	Indoor office	39.3 × 2.7 × 45.9	Cubicle partitions, doors, dry walls, elevator	NLOS	2.7–3.2	9.6–11.3	51.3–76.3	2.7–3.5	9.30–11.2
[77]	51–113	26–38	V–V	Indoor Library			NLOS	2.36–3.1893	3.78–8.87	52.63–100.7	0.96–4.18	3.7–7.86
This Work	2–24	28–38	V–V V–H	Indoor Corridor	2.63 × 1.4 × 30	Dry concretes and bricks, staircase, elevator, and office wooden doors	NLOS	2.82–3.46	1.68–10.48	65.11–87.82	0.97–3.31	0.78–2.94

Table abbreviations: Ref. = Reference, Dist. = Distance Range, Freq. range = Frequency range, Pol. = Polarization, Envi. = Environment, Dimen. = Dimension, Sce. = Scenario.

## Data Availability

Not applicable.
